# Determinants of Major Cardiovascular Risk Factors Among Participants of the South Carolina WISEWOMAN Program, 2009–2012

**DOI:** 10.5888/pcd11.140044

**Published:** 2014-09-04

**Authors:** Georges Joseph Nahhas, Virginie Daguise, Andrew Ortaglia, Anwar T. Merchant

**Affiliations:** Author Affiliations: Virginie Daguise, South Carolina Department of Health and Environmental Control, Columbia, South Carolina; Andrew Ortaglia, Anwar T. Merchant, University of South Carolina, Columbia, South Carolina.

## Abstract

**Introduction:**

Cardiovascular disease (CVD) is the leading cause of death among US women, accounting for 25% of all deaths in this population. Approximately 65% of these deaths occur in asymptomatic women. Hypertension, hypercholesterolemia, and diabetes mellitus (diabetes) are major risk factors for CVD and can be treated effectively if identified at an early stage.

**Methods:**

Data were available from 3,572 uninsured first-time female participants aged 40 to 65 years, referred by their health professional to the South Carolina Well-Integrated Screening and Evaluation for Women Across the Nation (SC WISEWOMAN), 2009–2012. All women completed a structured health-risk and behavior questionnaire. Anthropometric measures were recorded and data on clinical risk-factors were collected. Prevalence-ratios (PRs) were obtained by predictive multivariable log-linear modeling.

**Results:**

The prevalence of risk factors was 34.7% for uncontrolled hypertension, 9.3% for hypercholesterolemia, and 21% for diabetes. Prevalence of untreated hypertension was 15.6%; hypercholesterolemia, 8%; and diabetes, 4%. The greatest significant predictor of hypercholesterolemia was hypertension (PR = 4.37) and vice versa (PR = 2.39). The greatest significant predictors of diabetes were obesity (PR = 2.23), family history of diabetes (PR = 2.02), and hypercholesterolemia (PR = 1.85). Being obese (PR = 1.36), overweight (PR = 1.23), aged 60 years or more (PR = 1.26), and black (PR = 1.14) were significant predictors of having at least one CVD risk factor. Being black (PR = 1.09) was the only significant predictor of having comorbid conditions.

**Conclusion:**

Prevalence of uncontrolled CVD risk factors was high among participants in the SC WISEWOMAN program. These findings confirm that the program is reaching high-risk women who are in need of interventions to reduce their risk for CVD through lifestyle changes.

## Introduction

Cardiovascular disease (CVD) is the leading cause of death in the United States overall and among white and black women, accounting for 5 of 20 deaths among women in 2009 (1–3). Approximately 65% of these deaths occurred in asymptomatic women (4).

Hypertension (5,6), hypercholesterolemia (7), and hyperglycemia (8) are the 3 main risk factors for CVD. Although such conditions can be treated effectively (9), they may not be highly controlled in the US population in general and in older women in particular (10). Women of lower socioeconomic status have higher rates of CVD and live in an environment that promotes high-risk behavior (3). A national survey reported a doubling in awareness of CVD among women in the past decade (3,11), and another study showed a positive correlation between women’s awareness of CVD risk and taking a preventive action toward reducing it (12). More efforts, however, are needed in education and awareness campaigns to promote knowledge of CVD and ways to reduce its burden in highly susceptible populations (3,13,14).

South Carolina is a rural southern state with inadequate health resources and high reported rates of diabetes (12.1%) and stroke (3.7%) (15). In 2013, 5 of 46 South Carolina counties reported a shortage of health care providers (16). Under such conditions, it is crucial to explore state-wide patterns of distribution and treatment of CVD to provide appropriate case management and health care and to prevent further development of CVD risk factors among South Carolina residents.

Our study objective was to determine the prevalence of uncontrolled hypertension, hypercholesterolemia, and diabetes and to identify the predictors of these risk factors among women participating in the South Carolina Well-Integrated Screening and Evaluation for Women Across the Nation (SC WISEWOMAN) program for the first time from 2009 through 2012.

## Methods

### Study participants

**Figure 1:**
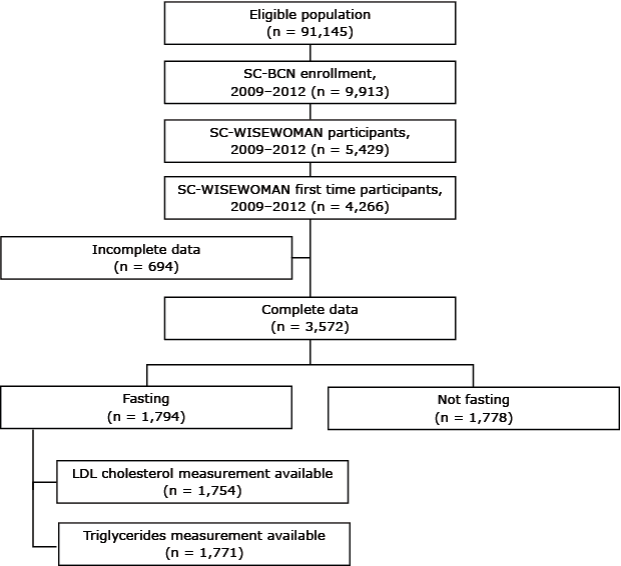
Selection process for participants in our study of major cardiovascular risk factors among women participating in the South Carolina WISEWOMAN program, 2009–2012. Abbreviations: SC BCN, South Carolina Best Chance Network; LDL, low-density lipoprotein.

In 2008 the South Carolina Department of Health and Environmental Control received funding from the Centers for Disease Control and Prevention (CDC) to implement the SC WISEWOMAN program (17), a program that provides screening for CVD risk factors (diabetes, obesity, hypercholesterolemia, hypertension) and referral to lifestyle interventions that provide counseling on healthy diets, physical activity, and smoking cessation. Eligibility criteria for SC WISEWOMAN are based on the framework provided by the state’s breast and cervical cancer screening program, the Best Chance Network (BCN), which is part of CDC’s National Breast and Cervical Cancer Early Detection Program. From May 2009 through June 2012 both the BCN and SC WISEWOMAN programs were available to 91,145 South Carolina aged 40 to 65. Women could enroll in both programs simultaneously at the health center where they received their health care if that center provided access to both programs. Women were eligible to participate in SC WISEWOMAN only if they had had breast and cervical cancer screening through the BCN. Of 91,145 South Carolina women eligible for BCN from May 2009 through June 2012, 9,913 were participating in that program; 5,429 of these women were also participating in SC WISEWOMAN. Of these, 4,266 were first-time SC WISEWOMAN participants (Figure 1). Our study group consisted of 3,572 of these first-time participants for whom complete cross-sectional data were available indicating that they were aged 40 to 65 years, were uninsured or underinsured, were living at or below 200% of the federal poverty level, and had received screening for breast and cervical cancer through the BCN. Participants were considered to be underinsured if their insurance covered hospitalization only. We recruited women from the general South Carolina population by different strategies, including posters, flyers, brochures, and word-of-mouth. Our study was reviewed and approved by the institutional review board of the South Carolina Department of Health and Environmental Control.

### Data collection

After participants provided consent to trained health professionals, they were administered a structured health-risk and behavior questionnaire as required by CDC. The provider (physician, nurse, or office assistant) filled out the questionnaire if the participant could not read or write. Information on sociodemographic and behavioral factors, personal medical history, and family medical history was collected. Clinical anthropometric measures were taken during a physical examination and included height and weight and systolic (SBP) and diastolic (DBP) blood pressure measured by using standard scales. Laboratory tests included total cholesterol, high-density lipoprotein (HDL) cholesterol, low-density lipoprotein (LDL) cholesterol, triglycerides, and blood glucose or blood hemoglobin A1c (HbA1c). Fasting status was recorded. LDL cholesterol and triglyceride levels were calculated only for participants who were fasting. Screenings were performed at federally qualified health centers. All centers contracted with Laboratory Corporation of America to perform blood-work procedures (total cholesterol, HDL cholesterol, LDL cholesterol, triglycerides, blood glucose, and HbA1c). SBP and DBP were measured according to the Seventh Report of the Joint National Committee on Prevention, Detection, Evaluation, and Treatment of High Blood Pressure guidelines (18).

### Study variables

Age was grouped in 3 categories: 49 years or younger, 50 to 59 years, and 60 years or older. Race was defined as either white or black. Educational level was categorized as high school graduate or equivalent, more than high school, and less than high school. Smoking status was defined by answering yes or no to the question, “Do you now smoke cigarettes every day, some days, or not at all?” Body mass index (BMI) was calculated as weight in kilograms divided by height in meters squared (kg/m^2^) and defined as underweight (<18.5 kg/m^2^), normal weight (25.0–29.9 kg/m^2^), overweight (18.5–24.9 kg/m^2^), or obese (≥30 kg/m^2^) (19). Clinically determined blood pressure was classified into 3 groups: prehypertension (SBP ≥120 mm Hg or DBP ≥80 mm Hg), normal (SBP <120 mm Hg and DBP <80 mm Hg) (20), and hypertension (SBP ≥140 mm Hg or DBP ≥90 mm Hg among nondiabetic women and SBP ≥130 mm Hg or DBP ≥80 mm Hg for women with diabetes) (21).

Total cholesterol level was defined as desirable (<200 mg/dL) or undesirable (≥200 mg/dL). HDL cholesterol was defined as desirable (>59 mg/dL) or undesirable (≤59 mg/dL). LDL cholesterol was defined as desirable (<130 mg/dL) or undesirable (≥130 mg/dL) (22). Triglycerides concentration was defined as desirable (<150 mg/dL) or undesirable (≥150 mg/dL) (23). Self-reported family history of heart attack was defined as yes or no to the question “Has a doctor, nurse, or other health professional ever told you that you had any of the following: heart attack (also called myocardial infarction), angina, coronary heart disease, or stroke?” We used the same question for diabetes, “Have you ever been told by a doctor, nurse, or other health professional that you have diabetes?” Family history questions were restricted to first-degree relatives (ie, parent, sibling, or child). Self-reported taking of medication for hypertension, hypercholesterolemia, or diabetes was also classified by answering yes or no to the question, “Has your father, brother, son, mother, sister, or daughter had a stroke or heart attack before age 55?” Presence of clinically determined hypercholesterolemia was defined as total cholesterol at or above 240 mg/dL. Presence of clinically determined type 2 diabetes was defined as fasting blood glucose above 125 mg/dL or nonfasting blood glucose above 199 mg/dL or if the patient’s medical record indicated a diagnosis of diabetes. Untreated hypertension (newly identified hypertension) was defined as women who answered no to the question “Are you taking any medicine prescribed by your doctor, nurse, or other health professional for your high blood pressure?” but had clinically determined hypertension. Uncontrolled cases of hypertension were defined as women with clinically determined high blood pressure who answered yes to the question “Are you taking any medicine prescribed by your doctor, nurse, or other health professional for your high blood pressure?” Treated and controlled hypertension was defined as women with no clinically determined high blood pressure who answered yes to the question, “Are you taking any medicine prescribed by your doctor, nurse, or other health professional for your high blood pressure?” Hypertension was defined as absent if a woman had no clinically determined hypertension and answered no to the question “Are you taking any medicine prescribed by your doctor, nurse, or other health professional for your high blood pressure?” We asked the same questions, substituting hypercholesterolemia or diabetes for hypertension, to assess participants’ status with respect to these 2 conditions.

### Statistical analysis

All computations and statistical analyses were performed by using SAS 9.3 (SAS Institute, Inc). Descriptive statistics were used to estimate prevalence of treatment and control of CVD risk factors (hypertension, hypercholesterolemia, and diabetes), self-reported family history of heart attack and diabetes, and clinically determined risk factors for CVD. The prevalence of each CVD risk factor was determined by each factor’s treatment and control, which was calculated as the sum of the proportions of those who were untreated, uncontrolled, and treated and controlled. To the best of our knowledge, only Ahluwalia et al. (24) have reported such classification of risk factor conditions for WISEWOMAN participants. The relationship between each of the 3 CVD risk factors, treated as outcome variables, and proposed predictors was assessed separately by using a log-linear model with the “log” link and Poisson distribution, which allows the calculation of prevalence ratios (PRs). The same modeling technique was used to determine significant predictors of having at least 1 CVD risk factor, and for having more than 1 versus only 1. After the multivariable regression full model was specified, backward elimination was implemented, and different models were assessed for goodness-of-fit by the likelihood ratio test. The full model included diabetes, hypertension, hypercholesterolemia, age, race, educational level, smoking status, family history of heart attack, family history of diabetes, and BMI. Predictors were considered to be significant if their elimination resulted in a significant likelihood ratio test (*P* < .05), irrespective of their individual significance in the model.

## Results

The mean age of the participants was 54 (standard deviation [SD] = 5.7), ranging from 40 through 65; approximately 50% were aged 50 to 59. Most (59%) were black. Most participants (49%) had a high school degree; 30% had more than high school, and 22%, less than high school. About one-third were current smokers. Family history of diabetes and heart attack was reported by 53% and 35% of the participants, respectively. The average BMI was 32.4 (SD = 7.6) and ranged from 18.5 to 72.0, with most women in the obese category (58%); none was underweight. The mean SBP was 130 mm Hg (SD = 18 mm Hg), ranging from 84 to 225 mm Hg, and the mean DBP was 80 mm Hg (SD = 10 mm Hg), ranging from 49 to 413 mm Hg; 55% were prehypertensive. The average total, fasting or nonfasting, cholesterol level was 201 mg/dL (SD = 43 mg/dL ); about half had cholesterol levels higher than normal. The mean HDL level was 57 mg/dL (SD = 17 mg/dL); about 13% had undesirable levels. The mean level of LDL cholesterol was 119 mg/dL (SD = 38 mg/dL) and was available for 1,754 women, and that of triglycerides was 121 mg/dL (SD = 80 mg/dL) and was available for 1,771 women; most had desirable levels of LDL cholesterol (66%) and triglycerides (77%) (Table 1).

**Table 1:** Distribution of Cardiovascular Risk Factors by Sociodemographic Characteristics Among First-Time Attendees (N = 3,572), South Carolina WISEWOMAN Program, 2009–2012

Characteristic	n (%)
**Age, y**	
≤49	955 (26.7)
50–59	1, 772 (49.6)
≥ 60	845 (23.7)
**Race**	
White	1,470 (41.1)
Black	2,102 (58.9)
**Education**	
Less than high school	773 (21.6)
High school graduate or equivalent	1,734 (48.6)
More than high school	1,065 (29.8)
**Current smoker**	
No	2,492 (69.8)
Yes	1,080 (30.2)
**Self-reported family history of heart attack**	
No	2,305 (64.5)
Yes	1,267 (35.5)
**Self-reported family history of diabetes**	
No	1,690 (47.3)
Yes	1,882 (52.7)
**Body mass index, kg/m^2^**	
Normal weight (25.0–29.9 kg/m^2^)	540 (15.1)
Overweight (18.5–24.9 kg/m^2^)	955 (26.7)
Obese (≥30.0 kg/m^2^)	2,077 (58.2)
**Blood pressure, mm Hg^a^**	
Normal	851 (23.8)
Prehypertension	1,968 (55.1)
Hypertension^a^	753 (21.1)
**Total cholesterol, mg/dL**	
Desirable (<200)	1,866 (52.2)
Undesirable (≥200)	1,706 (47.8)
**High-density lipoprotein cholesterol, mg/dL**	
Desirable (>59)	2,233 (87.5)
Undesirable (≤59)	447 (12.5)
**Low-density lipoprotein cholesterol^b^^, ^ c, mg/dL**	
Desirable (<130)	1,161 (66.1)
Undesirable (≥130)	594 (33.9)
**Triglycerides^b^^, ^ d, mg/dL**	
Desirable (<150)	1, 367 (77.2)
Undesirable (≥150)	404 (22.8)
**Type 2 diabetes**	
No	2, 695 (75.5)
Yes	877 (24.5)

a Normal systolic blood pressure (SBP) <120 and normal diasystolic blood pressure (DBP) <80; prehypertension, SBP ≥120 or DBP ≥80; hypertension, SBP ≥140 mm Hg, DBP ≥90 mm Hg for nondiabetic women; SBP ≥130 mm Hg or DBP ≥80 mm Hg for women with diabetes.

b Collected only for participants who were fasting (n = 1,794).

c Low-density lipoprotein cholesterol measurement was missing for 40 fasting participants.

d Triglyceride measurement was missing for 23 fasting participants.

**Figure 2:**
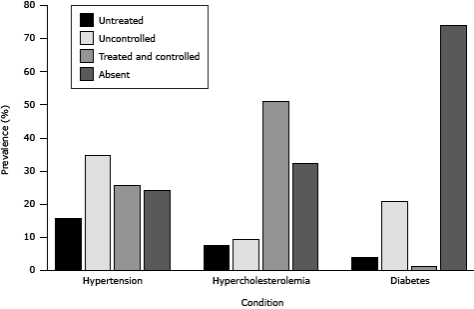
Prevalence of cardiovascular risk factors of first-time attendees (n = 3,572) of the South Carolina-WISEWOMAN, 2009–2012. **Table:** 

The prevalence of untreated diabetes was 4%, and that of uncontrolled diabetes, 21%. The prevalence of untreated hypertension was about 16% and that of uncontrolled hypertension, 35%. The prevalence of hypercholesterolemia was 8% for untreated and 9% for uncontrolled. The prevalence of hypertension was 76%; hypercholesterolemia, 68%; and diabetes, 26%, (Figure 2).

Disease	Condition	Prevalence (%)
Hypertension	Untreated	15.6
Hypertension	Uncontrolled	34.7
Hypertension	Treated and controlled	25.6
Hypertension	Absent	24.1
Hypercholesterolemia	Untreated	7.56
Hypercholesterolemia	Uncontrolled	9.27
Hypercholesterolemia	Treated and controlled	51.0
Hypercholesterolemia	Absent	32.2
Diabetes	Untreated	4.00
Diabetes	Uncontrolled	20.9
Diabetes	Treated and controlled	1.20
Diabetes	Absent	73.9

The significant predictors of type 2 diabetes were being older than 60 (PR = 1.35), being black (PR = 1.33), not having a high school diploma (PR = 1.26), having a family history of diabetes (PR = 2.02), being overweight (PR = 1.68) or obese (PR = 2.23), having hypercholesterolemia (PR = 1.85), and having hypertension (PR = 1.86) (Table 2). The significant predictors of hypertension were being black (PR = 1.12) and having hypercholesterolemia (PR = 2.39). The significant predictors of hypercholesterolemia were having hypertension (PR = 4.37), type 2 diabetes (PR = 1.13), and being obese (PR = 1.18) or overweight (PR = 1.12). Being aged 50 to 59 years (PR = 1.19) or over 60 years (PR = 1.26), black (PR = 1.14), overweight (PR = 1.23), or obese (PR = 1.36) were significant predictors of having at least 1 risk factor. However, the only significant predictor of having more than 1 CVD risk factor was being black (PR = 1.09) (Table 3) .

**Table 2:** Adjusted Predictors of 3 Baseline Cardiovascular Risk Factors Among First-Time Attendees (N = 3,572), South Carolina WISEWOMAN Program, 2009–2012^a^

Characteristic	Type 2 Diabetes^b^	Hypertension^c^	Hypercholesterolemia^d^
**Age, y**			
≤49	1 [Reference]	—e	1 [Reference]
50–59	1.07 (0.90–1.79)	—e	1.18 (1.07–1.31)
≥ 60	1.35 (1.12–1.62)	—e	1.21 (1.08–1.37)
**Race**			
Black	1.33 (1.15–1.54)	1.12 (1.04–1.22)	—e
White	1 [Reference]	1 [Reference]	—e
**Education**			
High school graduate or equivalent	0.99 (0.84–1.16)	—e	—e
More than high school	1 [Reference]	—e	—e
Less than high school	1.26 (1.06–1.51)	—e	—e
**Self-reported family history of diabetes**			
Yes	2.02 (1.75–2.33)	—e	—e
No	1 [Reference]	—e	—e
**BMI (kg/m^2^)**			
Normal weight (25–29.9 kg/m^2^)	1 [Reference]	—e	1 [Reference]
Overweight (18.5–24.9 kg/m^2^)	1.68 (1.23–2.29)	—e	1.12 (0.97–1.30)
Obese (≥ 30 Kg/m^2^)	2.23 (1.67–2.99)	—e	1.18 (1.03–1.35)
**Hypercholesterolemia**			
Yes	1.85 (1.47–2.32)	2.39 (2.16–2.65)	—e
No	1 [Reference]	1 [Reference]	—e
**Hypertension**			
Yes	1.86 (1.40–2.46)	—e	4.37 (3.69–5.17)
No	1 [Reference]	—e	1 [Reference]
**Type 2 Diabetes**			
Yes	—e	—e	1.13 (1.04–1.24)
No	—e	—e	1 [Reference]

Abbreviations: PR, prevalence ratio; CI, confidence interval; BMI, body mass index.

a All values are PR (95% CI). Adjusted for all the other variables in the model.

b Final prediction model was adjusted for age, race, education, self-reported family history of diabetes, BMI, hypercholesterolemia, and hypertension.

c Final prediction model was adjusted for race and hypercholesterolemia

d Final prediction model was adjusted for age, hypertension, and diabetes.

e Does not apply.

**Table 3:** Predictors of 3 Baseline Cardiovascular Risk Factors Among First-Time Attendees (N = 3,572), South Carolina WISEWOMAN Program, 2009–2012^a^

Characteristic	At Least 1 Cardiovascular Risk Factor^b^ (n = 3,572)	>1 Versus 1 Cardiovascular Risk Factor^c^ (n = 2,920)
**Age, y**		
≤49	1 [Reference]	—d
50–59	1.19 (1.09–1.30)	—d
≥60	1.26 (1.14–1.40)	—d
**Race**		
Black	1.14 (1.05–1.22)	1.09 (1.03–1.15)
White	1 [Reference]	1 [Reference]
**Body mass index, kg/m^2^**		
Normal weight (18.5–24.9)	1 [Reference]	—d
Overweight (25.0–29.9)	1.23 (1.08–1.40)	—d
Obese (≥30.0)	1.36 (1.20–1.52)	—d

Abbreviations: PR, prevalence ratio; CI, confidence interval.

a All values are PR (95% CI). Adjusted for all other significant variables in the model.

b Final prediction model was adjusted for age, race, and body mass index.

c Final prediction model was adjusted for race.

d Does not apply.

## Discussion

The prevalence of untreated and uncontrolled diabetes, hypertension, and hypercholesterolemia was high in this group of low-income, underinsured women in South Carolina. The prevalence of at least one of these risk factors was higher among women who were older, black, and overweight or obese. Women newly diagnosed (untreated) with hypertension, hypercholesterolemia, and type 2 diabetes were identified. SC WISEWOMAN participants had high rates of uncontrolled hypertension (35%) and type 2 diabetes (21%).

Of the 3,572 participants in SC-WISEWOMAN, 50% were aged 50 to 59 years, 59% were black, 31% were current smokers, and 78% had a high-school degree or higher. At initial screening, rates of high blood cholesterol (48%), hypertension (21%), and diabetes (25%) were much higher than those reported in the general South Carolina population (high blood cholesterol, 41%; hypertension, 34%; and diabetes, 11% (25).

A recent study of the same program implemented in West Virginia reported that among 733 participants, 84% were overweight, 44% were prehypertensive, and 45% had high blood cholesterol levels (24). Another study, which described the WISEWOMAN program of Nebraska, reported that of 10,739 women screened, 75% were overweight, 56% were prehypertensive, and 20% had high blood cholesterol levels (26). Our findings are in line with findings from West Virginia but depart in some instances from those reported in Nebraska, which could be because geographically proximal states could share similar culture and dietary habits.

Given the high prevalence of these 3 CVD risk factors, generalized linear modeling gave more robust standard errors and a more meaningful measure of association, the prevalence ratio (27). The greatest predictors of type 2 diabetes were obesity (PR = 2.23), hypercholesterolemia (PR = 1.85), and hypertension (PR = 1.86). Although educational level was not significant in the final model, its presence contributed to the prediction of the outcome and reduced the variance of all other predictors. Hypercholesterolemia was the major predictor of hypertension (PR = 2.39) and vice versa (PR = 4.37). Unsurprisingly, obesity (PR = 1.36) was the greatest predictor of having at least 1 CVD risk factor; however, when comparing comorbid conditions to a single condition only race remained significant (PR = 1.09).

Our study had some limitations. First, our sample was not random, which limited the generalizability of our results to the underlying population; however, this screening program was tailored to target women at higher risk of developing CVD. Second, not all participants were fasting, so measures that require fasting (LDL cholesterol and triglycerides) were available for only 1,794 participants. However, many were recruited on the spot at different health centers at different times of the day, and fasting could not be ensured. Third, no information was collected on physical activity and dietary habits, so we could not assess how those 2 factors could influence the different CVD risk factors discussed here. Fourth, in the absence of a measure of central obesity we could not evaluate metabolic syndrome (28).

This study also had some strengths. First, the SC WISEWOMAN program reached 3,572 women at increased risk of developing CVD and provided subsequent care. Second, it identified new (untreated) cases of hypertension, hypercholesterolemia, and diabetes. Third, we used predictive statistical modeling for which confounding is not a risk, and we reported the prevalence ratio, which approximates risk better than the odds ratio (27). Fourth, this is the first study to examine participants of the SC WISEWOMAN program. Further research should evaluate the racial disparity in distribution and prediction of CVD risk factors among the participants of SC WISEWOMAN program and the effectiveness of the provided lifestyle interventions over time.

This study emphasizes the need for preventive measures targeted at hypertension, hypercholesterolemia, and diabetes and strategies to promote a healthy lifestyle, given the high levels of untreated and uncontrolled CVD risk factors among women aged 40 years or older in South Carolina. Our results stress the importance of checking for comorbidities, especially among higher-risk populations, and confirm that the program is reaching high-risk women who are in need of interventions tailored to reduce their risk for CVD through lifestyle changes. The SC WISEWOMAN screening program is reaching out to those who are at high risk of developing CVD and should be expanded to reach a larger geographic area.
